# Routine vaccination coverage at ages 2 and 7, before, during, and after the COVID-19 pandemic: Results from the STARVAX surveillance system

**DOI:** 10.17269/s41997-024-00956-9

**Published:** 2024-10-26

**Authors:** Ahash Jeevakanthan, Sophia Roubos, Cindy Hong, Allison Hender, Morag Granger, Sazzadul Khan, Maaz Shahid, Shannon LeBlanc, Jeanine O’Connell, Nicolas L. Gilbert

**Affiliations:** 1 Public Health Agency of Canada, Ottawa, ON Canada; 2 Alberta Health, Edmonton, AB Canada; 3 Saskatchewan Health, Regina, SK Canada; 4 Manitoba Health, Winnipeg, MB Canada; 5 Department of Health, Government of New Brunswick, Fredericton, NB Canada; 6 Department of Health, Government of Yukon, Whitehorse, YT Canada; 7 École de Santé Publique de l’Université de Montréal, Montréal, QC Canada

**Keywords:** Vaccine, Measles, Vaccination coverage, Surveillance, Pertussis, Immunization registry, COVID-19, Impact of the pandemic, Routine immunizations, Publicly funded vaccines, Vaccin, Rougeole, Couverture pour les vaccins, Surveillance, Coqueluche, Registre d’immunisation, COVID-19, Impact de la pandémie, Immunisations de routine, Vaccins financés par l’état

## Abstract

**Objective:**

A decline in routine vaccination coverage in children has been observed after the onset of the COVID-19 pandemic, but it is unclear whether these declines were sustained over time. This analysis assessed changes in vaccination coverage at ages 2 and 7 years for routinely administered vaccinations before, during, and after the COVID-19 pandemic.

**Methods:**

Vaccination coverage was measured using yearly data from the Standardized Reporting on Vaccination (STARVAX) surveillance system between December 31, 2019, and December 31, 2023. Four provinces (Alberta, Saskatchewan, Manitoba, and New Brunswick) and one territory (Yukon) produced reports from their immunization registries and provided the Public Health Agency of Canada with the numbers of vaccinated 2-year-old and 7-year-old children. Population estimates from Statistics Canada and the Yukon Bureau of Statistics were used as the denominators.

**Results:**

There was a decline in vaccination coverage in 2023 compared to 2019. In 2-year-olds, coverage for one dose of the measles, mumps, and rubella (MMR) vaccine and four doses of the diphtheria, tetanus, and acellular pertussis (DTaP) vaccine decreased from 89.5% to 82.5% and from 79.9% to 72.1%, respectively. Among 7-year-olds, DTaP (up-to-date) and MMR (two doses) vaccination coverage decreased from 77.1% to 68.8% and 86.3% to 75.6%, respectively.

**Conclusion:**

These declines are of concern and suggest that health care professionals should provide parents with accurate information regarding vaccines and encourage them to have their children vaccinated and keep vaccinations up to date.

**Supplementary Information:**

The online version contains supplementary material available at 10.17269/s41997-024-00956-9.

## Résumé

**Objectif:**

Un déclin des couvertures vaccinales de routine chez les enfants a été observé après le début de la pandémie de COVID-19 mais on ne sait pas si ce déclin s’est poursuivi par la suite. Cette analyse a mesuré le changement dans les couvertures vaccinales de routine chez les enfants de deux et sept ans avant, pendant et après la pandémie de COVID-19.

**Méthodes:**

Les couvertures vaccinales ont été mesurées en utilisant les données annuelles du système de surveillance Standardisation des rapports sur la vaccination (STARVAX) du 31 décembre 2019 au 31 décembre 2023. Quatre provinces (l’Alberta, la Saskatchewan, le Manitoba et le Nouveau-Brunswick) et un territoire (le Yukon) ont produit des rapports à partir des données de leur registre d’immunisation et fourni à l’Agence de la santé publique du Canada les nombres d’enfants de deux et sept ans vaccinés. Les estimations de taille de population de Statistique Canada et du Bureau de la statistique du Yukon ont été utilisées comme dénominateurs.

**Résultats:**

Les couvertures vaccinales étaient plus basses en 2023 qu’en 2019. Chez les enfants de deux ans, les couvertures pour une dose de vaccin contre la rougeole, la rubéole et les oreillons (RRO) et pour quatre doses de vaccin contre la diphtérie, la coqueluche et le tétanos (DCaT) ont diminué respectivement de 89,5% à 82,5% et de 79,9% à 72,1%. Chez les enfants de sept ans, les couvertures pour les vaccins DCaT (vaccination à jour) et RRO (deux doses) ont diminué respectivement de 77,1% à 68,8% et de 86,3% à 75,6%.

**Conclusion:**

Ces baisses des couvertures vaccinales sont préoccupantes et suggèrent que les professionnels de la santé donnant des soins aux jeunes enfants devraient informer correctement les parents au sujet des vaccins et les encourager à faire vacciner leurs enfants et garder leur vaccination à jour.

## Introduction

High vaccination coverage is essential for the prevention and control of vaccine-preventable diseases (Anderson & May, 1990). This has been highlighted by the measles outbreak that occurred in Canada in the winter and spring of 2024 as 77 measles cases were reported between January 1 and May 18, 2024 (PHAC, 2024c) compared to 12 in 2023 (PHAC, 2024d) and 3 in 2022 (PHAC, 2023).

Even before the pandemic, Canada had not achieved its 95% coverage goal for most childhood vaccinations (Gilbert et al., 2017; PHAC, 2022). A decline in routine vaccination coverage in children has been observed after the onset of the COVID-19 pandemic. Analyses conducted in Ontario (Ji et al., 2022), Quebec (Kiely et al., 2022), and Alberta (MacDonald et al., 2022) revealed decreased coverage in 2020 compared to pre-pandemic levels. It is unclear whether these declines continued over time.

The childhood National Immunization Coverage Survey (cNICS), the survey currently used to monitor routine vaccination coverage in children at the national level, is conducted every 2 years. As a result, there is typically a 2-year delay between the reference date for which coverage is calculated and the availability of results because of the time required to collect, validate, process, and analyze the data.

To address these delays, the Public Health Agency of Canada (PHAC) and its provincial and territorial partners developed the Standardized Reporting on Vaccination (STARVAX) system. STARVAX was built upon the success and infrastructure of the Canadian COVID-19 Vaccination Coverage Surveillance System (CCVCSS), which was used to measure vaccination coverage across Canada during the COVID-19 vaccination campaign (PHAC, 2024b). Similar to the CCVCSS, STARVAX is a collaborative initiative between PHAC and provincial and territorial partners designed to leverage immunization registries and provide more timely and accurate insights into national vaccination coverage for routine immunizations in children.

The system operates by gathering standardized reports from participating provinces and territories. These reports detail the number of people vaccinated by age, sex, vaccine type, and dose rank. These numbers are then consolidated into a central PostgreSQL database where it is queried to calculate vaccination coverage of routine immunizations. Currently, four provinces (Alberta, Saskatchewan, Manitoba, and New Brunswick) and one territory (Yukon) report the number of vaccinated children and adolescents to STARVAX. Another province (Nova Scotia) reports for adolescents only.

Each of the provinces and territories has its own vaccination schedule (PHAC, 2024e), though they are mostly based on the national Canadian Immunization Guide (PHAC, 2024a). For instance, all provinces and territories administer the first dose of the measles-mumps-rubella (MMR) or measles-mumps-rubella-varicella (MMR-V) vaccine at 12 months of age, followed by a second dose at either 18 months or between 4 and 6 years of age. Additionally, most provinces and territories (including all those participating in STARVAX) administer four doses of the diphtheria, tetanus, and acellular pertussis-containing vaccines (DTaP) at 2, 4, 6, and 18 months of age, followed by a dose of the tetanus, reduced diphtheria, and reduced acellular pertussis-containing vaccine (Tdap) between 4 and 6 years of age.

This analysis was undertaken to assess changes in vaccination coverage at ages 2 and 7 for routinely administered vaccinations for years 2019, 2020, 2021, 2022, and 2023 (i.e., before, during and after the COVID-19 pandemic) with the intent to contribute to the assessment of the risk of resurgence of vaccine-preventable diseases, especially measles.

## Methods

This analysis included Alberta, Saskatchewan, New Brunswick, Manitoba, and Yukon. Using data from their immunization registries, they reported on aggregated numbers of 2-year-old and 7-year-old children vaccinated as of December 31, 2019, 2020, 2021, 2022, and 2023. For 7-year-olds, New Brunswick only reported for 2022 and 2023. To keep this population consistent over the years, coverage estimates for 7-year-olds excluded New Brunswick. Similarly, New Brunswick was excluded from the DTaP estimates at age 2 because data were not available for some years. Reporting jurisdictions used statistical software, R or SAS, to analyze their data.

All jurisdictions reported to PHAC using a standardized report form (available as supplemental material) with common definitions, and inclusion and exclusion criteria. PHAC used R Shiny to transform and extract data from these tables into a Postgres database and combined coverage estimates across jurisdictions because not all provinces and territories agreed for PHAC to report provincial/territorial-specific results.

Denominators were population estimates obtained from Statistics Canada for Alberta, Saskatchewan, New Brunswick, and Manitoba. Denominators for Yukon were population estimates from the Yukon Bureau of Statistics. Population estimates for provinces were calculated as of January 1st of each year, while Yukon’s population estimates were as of December 31st.

Coverage rates were calculated by dividing the number of children vaccinated by the relevant population estimate. For instance, vaccination coverage in 2-year-olds in 2022 was the number of vaccinated 2-year-old children as of December 31, 2022, divided by the estimated population size of this age group as of December 31st or January 1, 2023. Confidence intervals were not reported because coverage rates were calculated from the entire population of participating provinces and territories, and not from samples of the population. Standard errors, and the confidence interval derived from them, are estimates of the error introduced by making inferences from samples to entire source populations. Therefore, they are not applicable in this case.

## Results

**Fig. 1:**
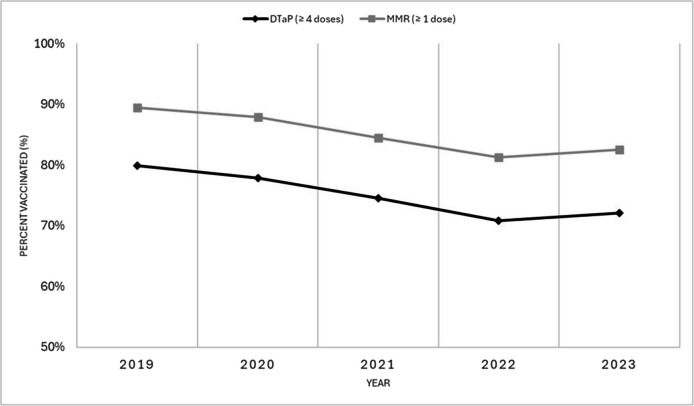
Coverage for diphtheria, tetanus, and acellular pertussis (DTaP) vaccines and measles, mumps, and rubella (MMR) vaccines in 2-year-old children in Alberta, Saskatchewan, Manitoba, New Brunswick (MMR only), and Yukon (combined), 2019 to 2023

Overall, vaccination coverage declined in 2023 compared to 2019. Specifically, in 2-year-olds, coverage for one dose of MMR and four doses of DTaP decreased from 89.5% to 82.5% and from 79.9% to 72.1%, respectively. When looking at yearly changes, between 2019 and 2022, sustained decreases in coverage were observed across all vaccines, followed by a slight increase or stabilization from 2022 to 2023 (Fig. 1). The most notable declines occurred during 2020 and 2021. Similar trends were observed for other vaccines at age 2, including varicella, rotavirus, polio, pneumococcal, and hepatitis B vaccines (Table S1).

**Fig. 2:**
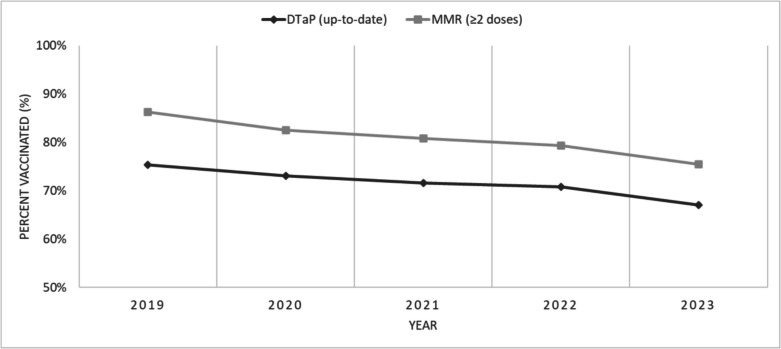
Coverage for diphtheria, tetanus, and acellular pertussis (DTaP) vaccines and measles, mumps, and rubella (MMR) vaccines in 7-year-old children in Alberta, Saskatchewan, Manitoba, and Yukon (combined), 2019 to 2023

Among 7-year-olds, coverage for up-to-date DTaP and two doses of MMR declined between 2019 and 2023 (Fig. 2). DTaP and MMR vaccination coverage went from 77.1% to 68.8% and 86.3% to 75.6%, respectively (Fig. 2). This decline in the older age group showed no apparent signs of recovery throughout the observed period (Fig. 2). Similar trends were observed for other routinely administered vaccines at age 7 (Table S2).

## Discussion

Our results show a decline in routine vaccination coverage was observed during the COVID-19 pandemic in Canada (Ji et al., 2022; Kiely et al., 2022; MacDonald et al., 2022) and elsewhere (Evans & Jombart, 2022; Yunusa et al., 2022), and that this persisted after the end of the pandemic. These findings are similar to those from a study of nine US states with immunization information systems: coverage at 2 years of age for MMR declined by 14.9% between the 2016 and 2020 birth cohorts, and the largest year-to-year decline was between the 2019 and 2020 birth cohorts (4.7%) (Treharne et al., 2024). This sustained decline in coverage is worrisome because of the risk of resurgence of vaccine-preventable disease, especially measles for which measured coverage was below the herd immunity threshold estimated to be 95% (Anderson & May, 1990). As mentioned earlier, Canada has already experienced a large measles outbreak in 2024 (PHAC, 2024c).

It is unclear whether there is a causal relationship between the observed decline in vaccination coverage and the COVID-19 pandemic. The pandemic and its associated vaccination campaign were accompanied by a large spread of misinformation, not only about COVID-19 vaccines, but about vaccines in general. However, two sequential surveys conducted in December 2020 and October–November 2021 on a panel of parents showed an increase in confidence over time in the safety and effectiveness of routine childhood vaccines (Rotolo et al., 2022), but there is no such data for 2022 and onward.

Efforts are being made to increase the vaccination coverage of infants and children. Initiatives to reduce barriers to immunization include expanding public health clinic hours into the evenings and in some cases weekends, contacting families whose children may be vaccine delayed, reminding families when their child is due for vaccines, and making clinics more accessible by providing outreach clinics. Additionally, registry teams are working to improve the accuracy of vaccination coverage estimates by enhancing data capture in provincial registries, including importing data from other sources. Working with communities, especially those who have lower vaccination coverage rates, allows immunizers to understand the issues and barriers families may face to get their children immunized and speak to vaccine hesitancy that may be present.

Future reporting cycles of STARVAX will tell us whether current and future efforts bring coverage in 2-year-olds and 7-year-olds back to their pre-pandemic levels. However, currently, birth cohorts cannot be followed over time using STARVAX, so any catch-up vaccinations for the birth cohorts that were found to have insufficient vaccination coverage cannot be captured. Additional investigation will be needed to answer this question. Moreover, further research is needed to understand the root causes of observed declines.

### Limitations of the study

This analysis has limitations. The completeness of registry data may be impacted by who vaccinates pre-school children (public health nurses only vs family doctors and paediatricians) and requirements to report vaccinations to registries, which varies between provinces and territories (Wilson et al., 2017). This limitation may result in the underestimation of immunization coverage. It may also be impacted by the implementation of new information systems in some participating jurisdictions. In addition, processes to identify and flag residents who moved outside the province or territory also vary. Some jurisdictions can link their registries with other government databases (e.g., health insurance) whereas others cannot. Incomplete identification of individuals who have moved after having been vaccinated would lead to overestimation of vaccination coverage.

## Conclusion

Together, these data suggest that STARVAX has enabled the measurement of vaccination coverage from provinces and territories in a timely manner to inform public health action. The declines observed in vaccination coverage are clearly of concern and health professionals providing care to children should take every opportunity to provide parents with accurate information and to encourage them to have their children vaccinated.

## Contributions to knowledge

What does this study add to existing knowledge?

- This study provides specific, empirical evidence of a sustained decline in routine vaccination coverage at ages 2 and 7 in selected Canadian provinces and a territory before, during, and after the height of the COVID-19 pandemic.
- It highlights the use of standardized reporting of vaccination coverage through immunization registries to monitor coverage at the national level in a timely manner.

What are the key implications for public health interventions, practice, or policy?

- Timely monitoring of vaccination coverage at the national level is necessary and possible through the use of registry-based surveillance systems like STARVAX.
- There is a need for catch-up vaccination activities, and for further investigation to measure catch-up.

## Supplementary Information

Below is the link to the electronic supplementary material.

Supplementary file1 (DOCX 45.4 KB)
