# Correction: Routine vaccination coverage at ages 2 and 7, before, during, and after the COVID-19 pandemic: Results from the STARVAX surveillance system

**DOI:** 10.17269/s41997-025-01043-3

**Published:** 2025-05-09

**Authors:** Ahash Jeevakanthan, Sophia Roubos, Cindy Hong, Allison Hender, Morag Granger, Sazzadul Khan, Maaz Shahid, Shannon LeBlanc, Jeanine O’Connell, Nicolas L. Gilbert

**Affiliations:** 1 Public Health Agency of Canada, Ottawa, ON Canada; 2 Alberta Health, Edmonton, AB Canada; 3 Saskatchewan Health, Regina, SK Canada; 4 Manitoba Health, Winnipeg, MB Canada; 5 Department of Health, Government of New Brunswick, Fredericton, NB Canada; 6 Department of Health, Government of Yukon, Whitehorse, YT Canada; 7 École de Santé Publique de l’Université de Montréal, Montréal, QC Canada

**Correction: Canadian Journal of Public Health**

10.17269/s41997-024-00956-9

This article has been updated to correct the reported declines in MMR and DTaP vaccination coverage in the abstract, results, and supplementary materials.

Among two-year-olds, MMR coverage was revised from 89.5% in 2019 and 82.5% in 2023 to 88.9% in 2019 and 84.3% in 2023. DTaP coverage was revised from 79.9% and 72.1% to 79.6% and 73.3%. Among seven-year-olds, MMR coverage was revised from 86.3% and 75.6% to 85.4% and 74.9%, and DTaP coverage from 77.1% and 68.8% to 77.3% and 68.2%.

In addition, the caption to Figure 1 was updated to remove 'MMR only' after Manitoba and New Brunswick, and Figures 1 and 2 were replaced (both versions appear below).
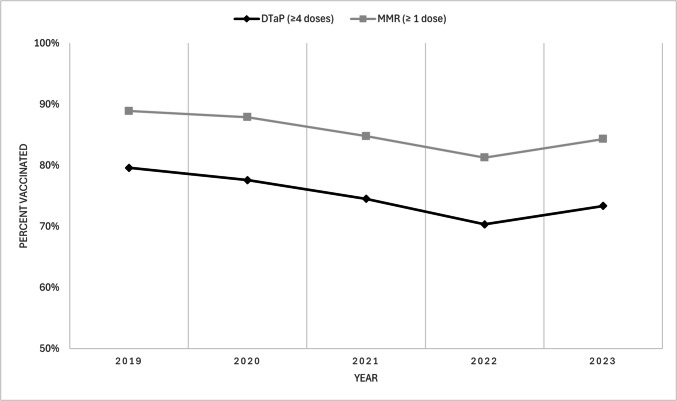



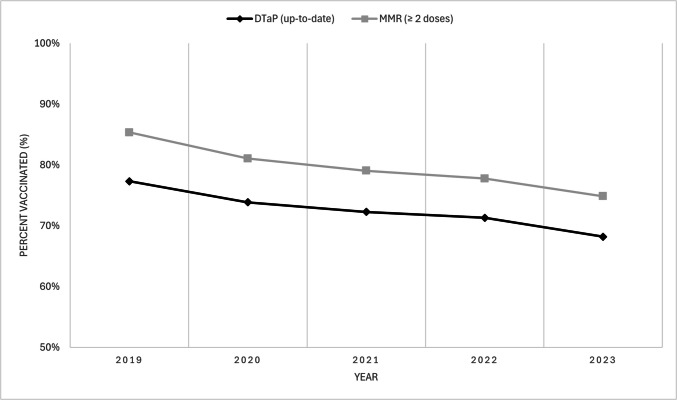


The supplementary material was replaced. The original supplementary material is posted here for clarity.

The Supplementary Information was also replaced on the original article to remove a note from Supplemental Table 1 (NB did not have appropriate data for Diphtheria, Tetanus and acellular Pertussis vaccinations for some reporting years and were therefore excluded for coverage at age 2). The original supplementary information is linked here for transparency.

## Supplementary Information

Below is the link to the electronic supplementary material.

Supplementary file1 (DOCX 48.9 KB)

