# Supplementary material for: Correction: Routine vaccination coverage at ages 2 and 7, before, during, and after the COVID-19 pandemic: Results from the STARVAX surveillance system
Source: Can J Public Health. 2025 May 9;116(4):609–11. doi: 10.17269/s41997-025-01043-3 (PMC12627295; doi:10.17269/s41997-025-01043-3)
Supplement: Supplementary file 1 — Supplementary file1 (DOCX 48.9 KB) [file 41997_2025_1043_MOESM1_ESM.docx]

## Supplemental material

| **Supplemental Table 1. Vaccination coverage over time age 2, recommended number of doses** | | | | | |
| --- | --- | --- | --- | --- | --- |
| **Antigens at age 2** | **2019-12-31** | **2020-12-31** | **2021-12-31** | **2022-12-31** | **2023-12-31** |
| Diphtheria, Tetanus and acellular Pertussis  (4 doses)^a^ | 79.6% | 77.6% | 74.5% | 70.3% | 73.3% |
| *Haemophilus influenzae* type b  (4 doses) | 79.6% | 77.6% | 74.5% | 70.3% | 73.4% |
| Hepatitis B (3 doses)^b^ | N/A | 79.6% | 88.1% | 82.9% | 86.1% |
| Meningococcal serogroup C (1-2 doses)^c^ | 85.3% | 85.0% | 82.2% | 78.0% | 80.2% |
| Measles, Mumps and Rubella  (1 dose) | 88.9% | 87.9% | 84.8% | 81.3% | 84.3% |
| Pneumococcal (3 doses) | 85.0% | 85.0% | 82.5% | 78.1% | 80.7% |
| Polio (3 doses) | 88.3% | 88.3% | 86.6% | 82.4% | 85.1% |
| Rotavirus (2 -3 doses)^d^ | 79.5% | 73.9% | 75.2% | 72.1% | 77.1% |
| Varicella (1 doses) | 88.3% | 87.5% | 84.5% | 81.0% | 84.0% |
| ^a^ NB did not have appropriate data for Diphtheria, Tetanus and acellular Pertussis vaccinations for some reporting years and were therefore excluded for coverage at age 2  ^b^ MB and SK follow a school program for Hepatitis B vaccination and were therefore excluded for coverage at age 2.  ^c^ Vaccination schedules vary among reporting provinces and territories. AB provides one dose at 2 and YT at 4 months for those at high risk of disease in addition to the dose provided by all at 12 months.  ^d^ Products vary among reporting provinces and territories. SK, NB, and YK provide RotaTeq (3 dose) whereas AB and MB provide Rotarix (2 doses).  **Supplemental Table 2. Vaccination coverage over time age 7, recommended number of doses** | | | | | |
| **Antigens at age 7** | **2019-12-31** | **2020-12-31** | **2021-12-31** | **2022-12-31** | **2023-12-31** |
| Diphtheria, Tetanus and acellular Pertussis  (up-to-date)^a^ | 77.3% | 73.9% | 72.3% | 71.3% | 68.2% |
| Measles, Mumps and Rubella (2 doses: fully vaccinated for measles and mumps) | 85.4% | 81.1% | 79.0% | 77.8% | 74.9% |
| Measles, Mumps and Rubella (1 dose: fully vaccinated for rubella) | 93.9% | 92.1% | 92.0% | 90.2% | 87.5% |
| Polio (3 doses) | 91.7% | 90.4% | 90.7% | 89.0% | 85.3% |

^a^ Up- to-date according to provincial and territorial recommendations
